# The Roles of Parathyroid Hormone-Like Hormone during Mouse Preimplantation Embryonic Development

**DOI:** 10.1371/journal.pone.0040528

**Published:** 2012-07-13

**Authors:** Lei Guo, Shu-Tao Qi, De-Qiang Miao, Xing-Wei Liang, Hui Li, Xiang-Hong Ou, Xin Huang, Cai-Rong Yang, Ying-Chun Ouyang, Yi Hou, Qing-Yuan Sun, Zhiming Han

**Affiliations:** 1 State Key Laboratory of Reproductive Biology, Institute of Zoology, Chinese Academy of Sciences, Beijing, China; 2 Graduate School, Chinese Academy of Sciences, Beijing, China; 3 Center of Reproductive Medicine, Department of Obstetrics and Gynecology, Nanfang Hospital, Southern Medical University, Guangzhou, China; University of Connecticut, USA, United States of America

## Abstract

Parathyroid hormone-like hormone (PTHLH) was first identified as a parathyroid hormone (PTH)-like factor responsible for humoral hypercalcemia in malignancies in the 1980s. Previous studies demonstrated that PTHLH is expressed in multiple tissues and is an important regulator of cellular and organ growth, development, migration, differentiation, and survival. However, there is a lack of data on the expression and function of PTHLH during preimplantation embryonic development. In this study, we investigated the expression characteristics and functions of PTHLH during mouse preimplantation embryonic development. The results show that *Pthlh* is expressed in mouse oocytes and preimplantation embryos at all developmental stages, with the highest expression at the MII stage of the oocytes and the lowest expression at the blastocyst stage of the preimplantation embryos. The siRNA-mediated depletion of *Pthlh* at the MII stage oocytes or the 1-cell stage embryos significantly decreased the blastocyst formation rate, while this effect could be corrected by culturing the *Pthlh* depleted embryos in the medium containing PTHLH protein. Moreover, expression of the pluripotency-related genes *Nanog* and *Pou5f1* was significantly reduced in *Pthlh*-depleted embryos at the morula stage. Additionally, histone acetylation patterns were altered by *Pthlh* depletion. These results suggest that PTHLH plays important roles during mouse preimplantation embryonic development.

## Introduction

Parathyroid hormone-like hormone (PTHLH), also known as parathyroid hormone-related protein (PTHrP), was first identified as a parathyroid hormone (PTH)-like factor responsible for humoral hypercalcaemia in malignancies in the 1980s [1]. Moniz *et al*. reported for the first time that PTHLH was expressed during normal human fetal development [2]. Unlike PTH, PTHLH expression was observed in many fetal tissues and adult tissues, even in the absence of hypercalcaemia [3], [4]. PTHLH has multiple physiological functions, including the regulation of morphogenesis, cell proliferation and differentiation, and transplacental calcium transport [5]. *Pthlh* null mice are characterized by chondrodysplasia, accelerated chondrocyte differentiation, endochondral ossification, and lethality minutes following birth [6]. *Pth1r* ^(−/−)^ mice demonstrate dysplastic long bone formation and early lethality, either in utero or within one to two days following birth [7].

Although many studies have reported that PTHLH is expressed in multiple tissues and plays multiple functions, few studies have investigated its expression characteristics and biological functions during preimplantation embryonic development. Van de Stolpe *et al*. reported that PTHLH is an endogenous inducer of parietal endoderm differentiation in the preimplantation mouse embryos, thus constituting the first identified example of an embryonic inducer in preimplantation mammalian development and the earliest hormone receptor system involved in embryogenesis defined to date [8]. They also showed for the first time that in the preimplantation mouse embryos, PTHLH was detected from the late morula stage onwards primarily in developing trophectoderm cells [8]. However, there was no functional study on the preimplantation embryonic development other than differentiation. Hereafter, Nowak *et al*. reported that PTHLH (1–34) and PTHLH (1–141) had no effect on the incidence of blastocyst formation in mouse preimplantation embryonic development [9]. Watson *et al*. reported that the supplementation of serum-free cSOFMaa oocyte maturation medium with PTHLH (1–141) resulted in a concentration-dependent increase in the development of bovine zygotes up to the blastocyst stage *in vitro* [10]. Both studies investigated the effects of exogenous PTHLH on preimplantation embryonic development, but the biological functions of endogenous PTHLH during mouse preimplantation embryonic development are yet unclear. Based on the extensive roles of PTHLH during the development and the expression of PTHLH in the preimplantation mouse embryos, we hypothesized that the endogenous PTHLH might play important roles during mouse preimplantation embryonic development by affecting the developmental capacity of mouse preimplantation embryos, the quality of blastocyst and the transcription of the preimplantation embryos.

In this study, for the first time, the expression characteristics of *Pthlh* was investigated, and siRNA-mediated depletion of *Pthlh* was used to investigate the involvement of endogenous PTHLH in mouse preimplantation embryonic development. The effects of down-regulation of this gene on developmental capacity, the expression of downstream or functionally related genes and histone acetylation dynamics were also examined.

## Results

### *Pthlh* Expression during Mouse Preimplantation Embryonic Development

**Figure 1:**
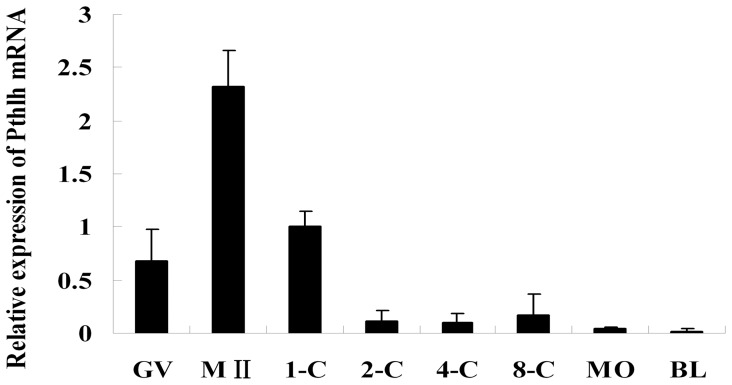
*Pthlh* expression in mouse oocytes and preimplantation embryos. Quantitative real-time PCR was applied to detect the expression of *Pthlh* in GV oocytes, MII oocytes, and in embryos at the 1-cell (1-C), 2-cell (2-C), 4-cell (4-C), 8-cell (8-C), morula (MO) and blastocyst (BL) stages. Transcripts levels were normalized against *Gapdh* expression. Data are presented as means ± SEM.

The expression of *Pthlh* in mouse oocytes and preimplantation embryos was examined using quantitative real-time PCR. The results show that *Pthlh* is expressed in mouse oocytes and preimplantation embryos at all developmental stages, with the highest level observed at the MII stage of the oocytes. *Pthlh* expression decreased dramatically following the 1-cell stage and reached its lowest level at the blastocyst stage (Fig. 1).

### Effects of *Pthlh* Depletion on Mouse Preimplantation Embryonic Development

**Figure 2:**
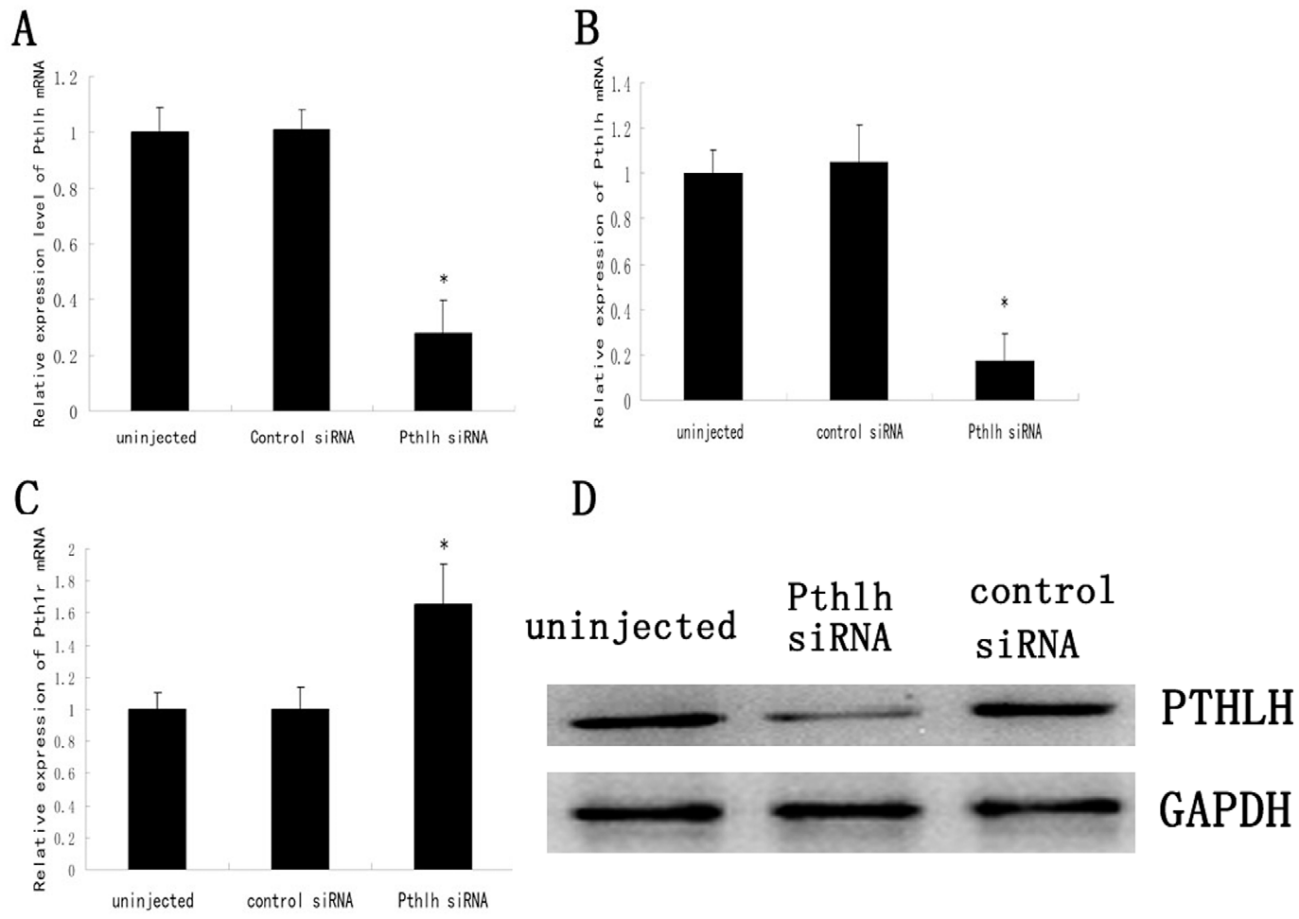
The effects of *Pthlh* siRNA injection on the expression of *Pthlh*, PTHLH and *Pth1r* in mouse preimplantation embryos. A) Quantitative real-time PCR was applied to detect the expression of *Pthlh* in the *Pthlh* siRNA-injected ICSI embryos at the 2-cell stage. Transcripts levels were normalized against *Gapdh* expression. Data are presented as means ± SEM. *p<0.01. B) *Pthlh* expression level in 2-cell embryos 24 h post-injection. Data are presented as means ± SEM. *p<0.01. C) *Pth1r* expression level in 2-cell embryos 24 h post-injection. Data are presented as means ± SEM. *p<0.01. D) Western blot results of PTHLH in the *Pthlh* siRNA-injected group and the control groups 24 h post-injection, GAPDH was used as the loading control.

The effects of *Pthlh* depletion on early mouse preimplantation embryonic development were assessed by introducing *Pthlh* siRNA into the oocytes at the MII stage and the preimplantaion embryos at the 1-cell stage. To ensure the efficiency of the siRNA-mediated knockdown prior to further studies, the expression levels of *Pthlh* were examined using quantitative real-time PCR and Western blot. The results showed that *Pthlh* expression level was decreased by 72.1% in the *Pthlh* siRNA-injected ICSI embryos compared with the control embryos at the 2-cell stage (Fig. 2A). The results also showed that the expression of *Pthlh* was reduced by 84.7% in the *Pthlh* siRNA-injected embryos compared with those from the control groups (100.0%) (*P<*0.01) at the 2-cell stage (Fig. 2B), and the protein level in the *Pthlh* siRNA-injected group also decreased compared with the control groups (Fig. 2D). However, compared with the control groups, the *Pth1r* expression level was increased in the *Pthlh* siRNA-injected group (100.0% *vs* 165.2%) (*P<*0.01) (Fig. 2C).

To investigate the effects of *Pthlh* depletion on blastocyst formation, 9 experiments were performed. The developmental rate and cell number at the blastocyst stage were examined. The *Pthlh* siRNA-injected and the control embryos were cultured *in vitro* until the control embryos developed to the expanded blastocyst stage. Notably, 51.7% embryos in the control siRNA-injected ICSI group and 52.5% of the uninjected ICSI embryos developed to the blastocyst stage, while only 24.5% embryos in the *Pthlh* siRNA-injected ICSI group developed to the blastocyst stage (P<0.01) (Table 1). Additionally, 83.1% of the control siRNA-injected embryos and 85.7% of the uninjected embryos developed to the blastocyst stage, whereas only 38.8% of the *Pthlh* siRNA-injected embryos developed to the blastocyst stage (P<0.01), and 49.0% of the *Pthlh* siRNA-injected embryos arrested at the morula stage (P<0.01) (Table 2, Table S1). The total cell number of the *Pthlh*-depleted embryos at the blastocyst stage (59.9±2.7, n = 21) was not significantly different from that of the control siRNA-injected group (63.3±2.7, n = 19) and the uninjected group (63.8±2.9, n = 20).

**Table 1:** Effect of *Pthlh* siRNA on development of mouse ICSI embryos.

Treatment	No. of embryos (No. exp)	No. of blastocysts (%)
uninjected	80 (3)	42 (52.5)
Control siRNA	87 (3)	45 (51.7)
*Pthlh* siRNA	102 (3)	25 (24.5)*

* p<0.01 compared with the control groups.

**Table 2:** Embryonic development following the microinjection of *Pthlh* siRNA into mouse embryos at the 1-cell stage.

	No. of embryos (%)					
Treatment	Total	2-c	4-c	8-c	Morula	Blastocyst
uninjected	210	3 (1.4)	7 (3.4)	4 (1.9)	16 (7.6)	180 (85.7)
Control siRNA	308	13 (4.2)	12 (3.9)	4 (1.3)	23 (7.5)	256 (83.1)
Pthlh siRNA	361	16 (4.4)	17 (4.7)	11 (3.1)	177 (49.0)*	140 (38.8)*

* p<0.01 compared with the control groups.

### *Pthlh* Depletion Down-regulates the Expression of *Pou5f1* and *Nanog*

**Figure 3:**
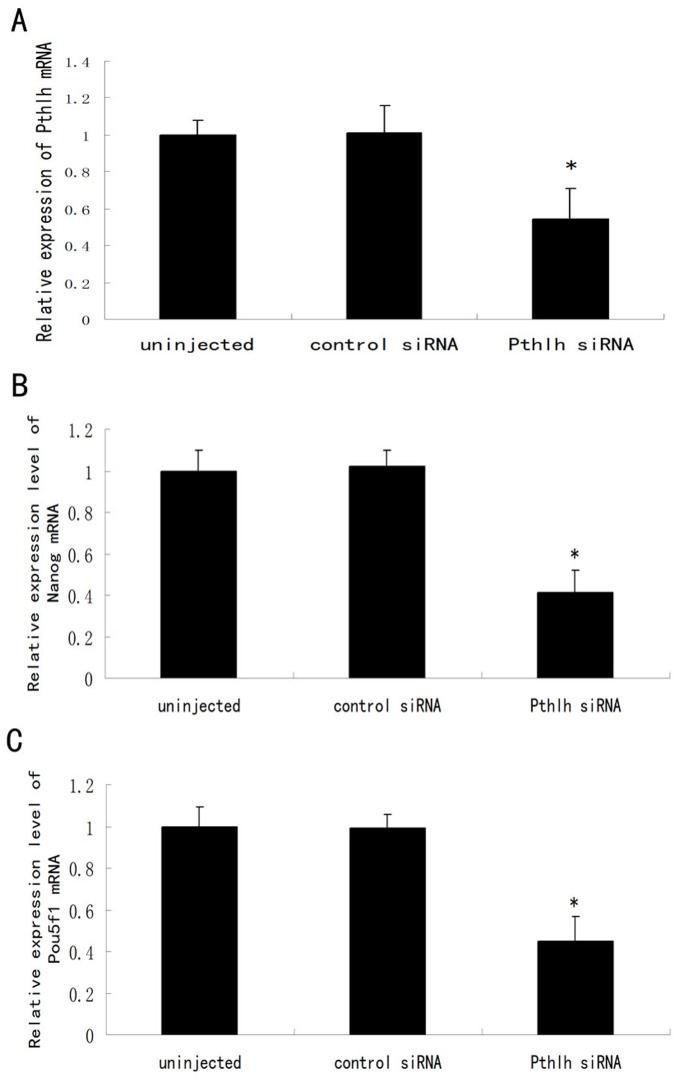
The effects of *Pthlh* siRNA injection on the expression of *Pthlh* (A), *Nanog* (B) and *Pou5f1* (C) in mouse morula stage embryos. The uninjected embryos and the control siRNA-injected embryos were used as controls. Data are presented as means ± SEM. *p<0.01.

**Figure 4:**
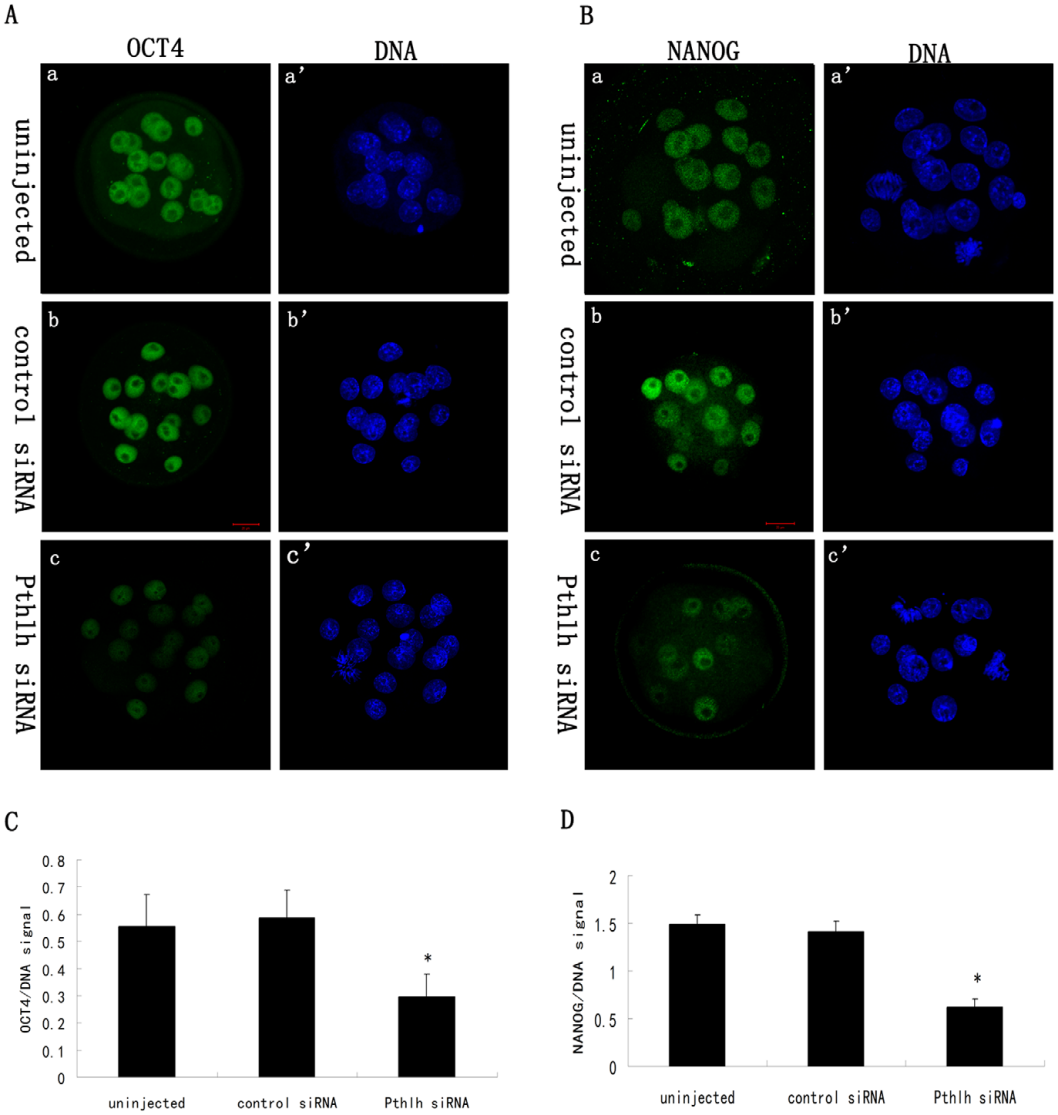
The effects of *Pthlh* siRNA injection on OCT4 and NANOG expression in mouse morula stage embryos. A) Staining pattern of OCT4 in the uninjected (a-a’), the control siRNA-injected (b-b’) and the *Pthlh* siRNA-injected (c-c’) embryos; B) Staining pattern of NANOG in the uninjected (a-a’), the control siRNA-injected (b-b’) and the *Pthlh* siRNA-injected (c-c’) embryos; C) Quantification of OCT4/DNA signal intensity in the *Pthlh* siRNA-injected and the control embryos (n = 14); D) Quantification of NANOG/DNA signal intensity in the *Pthlh* siRNA-injected and the control embryos (n = 13). OCT4/NANOG, green; DNA, blue. Bar  = 20 µm. Data are presented as means ± SEM. *P<0.01.

To investigate the effects of *Pthlh* depletion on the expression of the pluripotency-related genes *Nanog* and *Pou5f1* during mouse preimplantation embryonic development, quantitative real-time PCR and immunofluorescence staining were performed. Quantitative real-time PCR results showed that the expression levels of *Pthlh*, *Pou5f1* and *Nanog* were reduced by 50.3%, 55.0% and 59.3%, respectively, in the *Pthlh* siRNA-injected group compared with the control groups (100.0%) (P<0.01) at the morula stage (60 h post-siRNA injection) (Fig. 3). Decreased expression of OCT4 and NANOG was also demonstrated by immunofluorescence staining at the morula stage (Fig. 4, Fig. S1).

### Effects of *Pthlh* Depletion on the Histone Acetylation of Mouse Preimplantation Embryos

**Figure 5:**
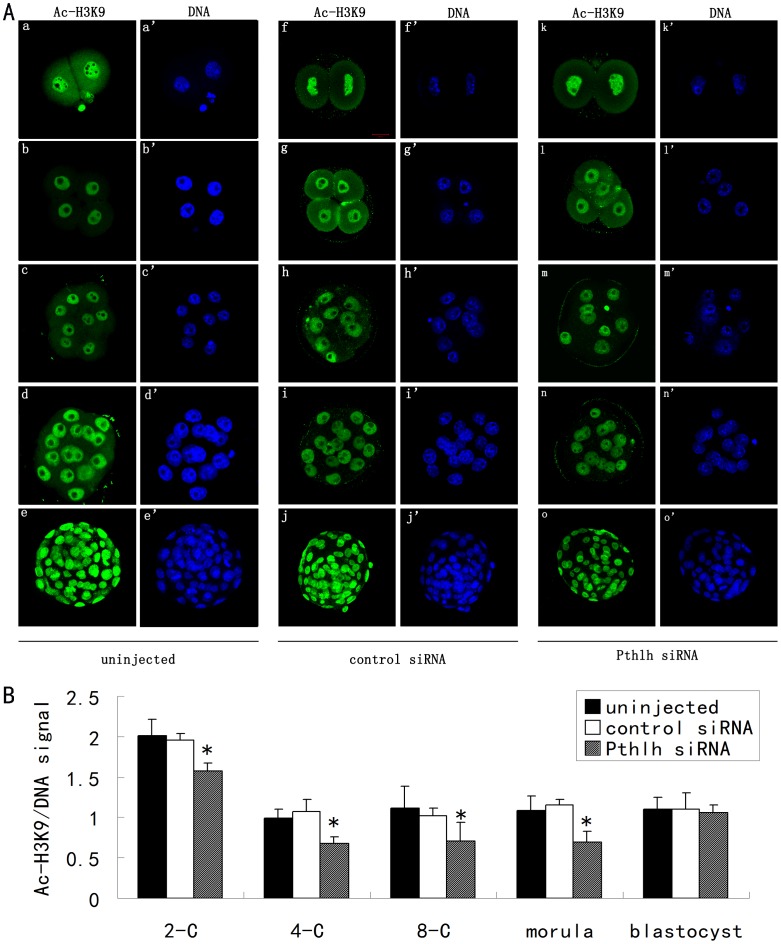
The effects of *Pthlh* siRNA injection on the acetylation of lysine 9 on histone H3 (Ac-H3K9) in mouse preimplantation embryos. A) The staining pattern of Ac-H3K9 in the uninjected (a-a’ to e-e’), the control siRNA-injected (f-f’ to j-j’) and the *Pthlh* siRNA-injected (k-k’ to o-o’) embryos at the 2-cell stage (a-a’, f-f’ and k-k’); the 4-cell stage (b-b’, g-g’ and l-l’); the 8-cell stage (c-c’, h-h’ and m-m’); the morula stage (d-d’, i-i’ and n-n’) and the blastocyst stage (e-e’, j-j’ and o-o’). Ac-H3K9, green; DNA, blue. Bar  = 20 µm. B) Quantification of Ac-H3K9/DNA signal intensity in the *Pthlh* siRNA-injected and the control embryos (n = 12). Data are presented as means ± SEM. *P<0.05.

**Figure 6:**
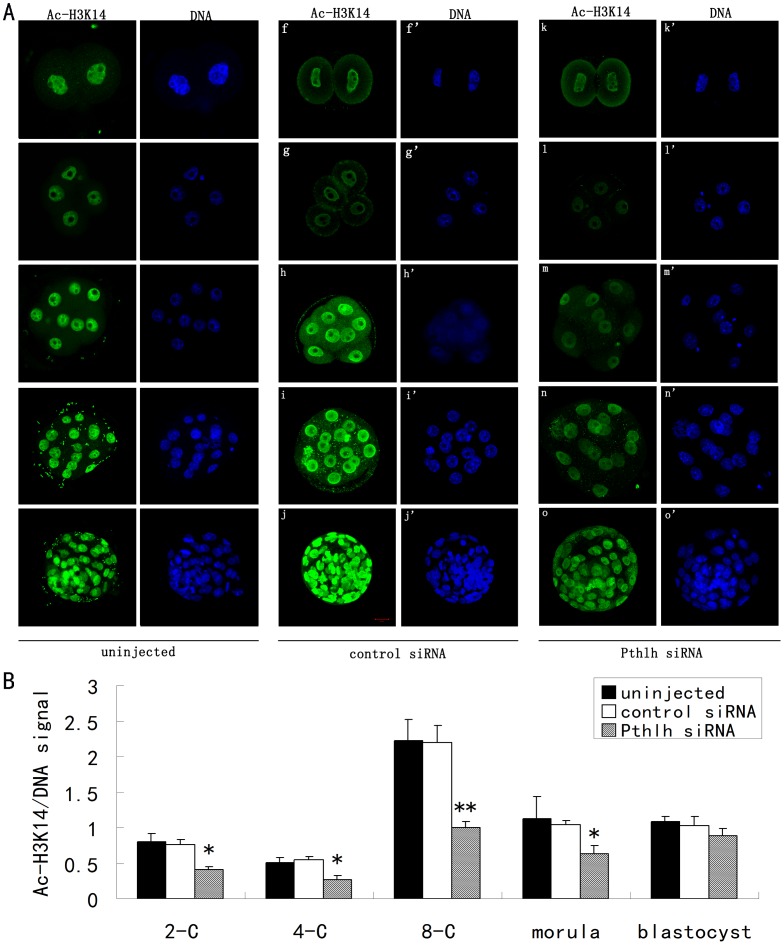
The effects of *Pthlh* siRNA injection on the acetylation of H3K14 (Ac-H3K14) **in mouse preimplantation embryos.** A) The staining pattern of Ac-H3K14 in the uninjected (a-a’ to e-e’), the control siRNA-injected (f-f’ to j-j’) and the *Pthlh* siRNA-injected (k-k’ to o-o’) embryos at the 2-cell stage (a-a’, f-f’ and k-k’); the 4-cell stage (b-b’, g-g’ and l-l’); the 8-cell stage (c-c’, h-h’ and m-m’); the morula stage (d-d’, i-i’ and n-n’) and the blastocyst stage (e-e’, j-j’ and o-o’). Ac-H3K14, green; DNA, blue. Bar  = 20 µm. B) Quantification of Ac-H3K14/DNA signal intensity in the *Pthlh* siRNA-injected embryos and the control embryos (n = 12). Data are presented as means ± SEM. *P<0.05, **P<0.01.

Immunofluorescence staining showed the following changes in histone acetylation of preimplantation embryos: the acetylation of lysine 9 on histone H3 (H3K9) and the acetylation of lysine 14 on histone H3 (H3K14) were both significantly decreased at the 2-cell, 4-cell, 8-cell and morula stages (Fig. 5, Fig. 6, Fig. S2, Fig. S3).

### Effects of PTHLH (1–141) on the Development of Embryos Injected with *Pthlh* siRNA

To investigate whether the exogenous target protein can improve the blastocyst development of the embryos injected with *Pthlh* siRNA, the PTHLH (1–141) was synthesized *in vitro*. After *Pthlh* siRNA injection, the embryos were cultured in KSOM^AA^ with or without 10 ng/ml PTHLH (1–141). 76.8% of embryos cultured in KSOM^AA^ with 10 ng/ml PTHLH (1–141) reached the blastocyst stage (Table 3), showing that the protein PTHLH (1–141) could improve the blastocyst development of the embryos injected with *Pthlh* siRNA.

**Table 3:** Effect of PTHLH (1–141) on development of mouse embryos injected with *Pthlh* siRNA.

Treatment	No. of embryos (No. exp)	No. of blastocysts (%)
KSOM^AA^	43 (3)	17 (39.5)
KSOM^AA^+10 ng/ml PTHLH (1–141)	56 (3)	43 (76.8)*

* p<0.01 compared with the control group.

## Discussion

In the present study, we determine for the first time that *Pthlh* is expressed in mouse oocytes and preimplantation embryos at all developmental stages and PTHLH plays important roles during mouse preimplantation embryonic development.

Before the functional studies of endogenous PTHLH during mouse preimplantation embryonic development were performed, the expression characteristics of *Pthlh* were investigated by quantitative real-time PCR. We observed the expression of *Pthlh* in mouse oocytes and preimplantation embryos at all developmental stages, with the highest level observed at the MII stage of the oocytes and the lowest level at the blastocyst stage of the preimplantation embryos. Given that the expression level of *Pthlh* decreased dramatically after the 1-cell stage, we examined the effects of *Pthlh* knockdown on preimplantation embryonic development by performing the siRNA-mediated *Pthlh* depletion at the MII stage oocytes and 1-cell stage embryos. Our data clearly indicated that the expression of *Pthlh* could be efficiently knocked down using siRNA mediated gene silencing in early preimplantation embryos. Further functional studies of PTHLH during mouse preimplantation embryonic development were performed based on the successful knockdown.

First, we investigated the effects of PTHLH on blastocyst formation. The formation of the blastocyst is an important landmark in the early developmental axis [11]. Blastocyst formation is often used as a criterion for developmental competence in *in vitro* embryo production systems. In the present study, *Pthlh* depletion was used to examine the role of PTHLH during mouse early embryonic development, especially blastocyst formation. The siRNA-mediated depletion of *Pthlh* at both MII oocytes and 1-cell embryos affected the blastocyst formation of mouse ICSI or fertilized embryos significantly. Nowak *et al*. reported that exogenous PTHLH (1–141) had no effect on the incidence of blastocyst formation in mouse preimplantation embryonic development [9]. However, we found that the PTHLH (1–141) had positive effect on the blastocyst formation rate of *Pthlh*-depleted embryos, which suggest that the lack of endogenous PTHLH can be corrected by exogenous PTHLH. The significantly decreased blastocyst formation rate of *Pthlh*-depleted embryos and the fact that PTHLH (1–141) could increase the blastocyst formation rate of the *Pthlh*-depleted embryos indicate that PTHLH is required for blastocyst formation.

Many transcription factors are required for reprogramming during mouse preimplantation embryonic development. The pluripotent state of the early embryo is established and maintained *in vivo* by a transcriptional network driven by a number of core regulatory genes, including *Oct4/Pou5f1* and *Nanog*. Blastocyst formation shows the segregation of the two cell lineages in mammalian preimplantation embryos, the inner cell mass (ICM) and trophectoderm (TE). As the important regulators of pluripotency in mammalian embryos, both *Oct4* and *Nanog* play important roles in the formation of the ICM. OCT4 is the earliest expressed transcription factor known to be crucial in murine preimplantation embryonic development [12], [13]. *Nanog* ^(−/−)^ embryos do not develop beyond implantation [14], and NANOG specifically demarcates the nascent epiblast [15], whereas ICM without *Nanog* cannot differentiate into primitive endoderm [14]. In the present study, the significantly decreased expression of *Nanog* and *Pou5f1* in *Pthlh*-depleted embryos indicates that *Pthlh* depletion can affect the preimplantation embryonic development by decreasing the expression of the pluripotency-related genes. There is no evidence showed that how the PTHLH affects the transcription factors (*Oct4/Pou5f1* and *Nanog*) in any cell types. Previous studies showed that the PTHLH can play roles through a complex network of signaling pathways involving cAMP, PKC and ERK/MAPK pathways [16]. Therefore, we speculated that the PTHLH might affect the expression of the pluripotency-related genes indirectly through those pathways during preimplantation embryonic development, but the mechanism needs further investigation.

Apart from the transcriptional network, epigenetic modifications play critical roles in mammalian embryo development. The acetylation of lysine residues on the tails of histones H3 and H4 is a reversible process that plays critical roles in maintaining higher-order chromatin structure and in regulating various chromatin processes, including transcription, DNA repair and replication. In the present study, the dynamic changes in the acetylation of different lysine residues on core histones H3 (H3K9 and H3K14) in *Pthlh*-depleted mouse embryos demonstrated that PTHLH can affect histone acetylation modification during preimplantation embryonic development. Although there is no evidence showed that the PTHLH exerts the direct effects on histone acetylation during preimplantation embryonic development, previous studies reported that the PTHLH exerts direct effects on histone deacetylase 4 (HDAC4) [17], [18]. Since the activity of histone deacetylase can be affected by PTHLH, the histone acetylation can be affected as a result during preimplantation embryonic development.

In the present study, our data provide the first evidence of the functions of endogenous PTHLH during mouse preimplantaion embryonic development. PTHLH influences blastocyst formation, *Nanog* and *Pou5f1* expression and the histone acetylation in mouse preimplantation embryos, indicating that *Pthlh* plays very important roles in the development of mouse preimplantation embryos.

## Materials and Methods

All chemicals and media were purchased from Sigma Chemical Company (St. Louis, MO) except for those specifically mentioned.

### Ethics Statement

Mice care and handling were conducted in accordance with the Animal Research Committee guidelines of the Institute of Zoology, Chinese Academy of Sciences. The institute does not issue a number to any animal study, but there is an ethical committee to guide animal use. Each study requires the permit to use animals from the committee, and this study was approved by the Animal Research Committee of the Institute of Zoology, Chinese Academy of Sciences. The animal facility must get licensing from the experimental animal committee of Beijing city. The animal handling staff (including each post-doc and Ph.D. student) must be trained before using animals. Mice were housed in a temperature-controlled room with proper darkness-light cycles, fed with a regular diet, and maintained under the care of the Laboratory Animal Unit, Institute of Zoology, Chinese Academy of Sciences. The mice were sacrificed by cervical dislocation. The only procedure performed on the dead animals is the collection of oocytes or embryos from the ovary or the oviduct.

### Collection of Mouse Oocytes and Embryos

Immature germinal vesicle (GV) stage oocytes were collected from 6- to 12-week-old B6D2F1 female mice 40 hours after they were injected with 5 IU of pregnant mare serum gonadotropin (PMSG). To obtain metaphase II (MII) stage oocytes, female mice were injected with 5 IU of PMSG followed 48 h later by 5 IU of human chorionic gonadotropin (hCG). To obtain fertilized embryos, females were superovulated by injection of 5 IU of PMSG, injected 48 h later with 5 IU of hCG, and then mated (males >10 weeks of age) [19], [20], [21]. Superovulated MII oocytes were obtained 14 hours following hCG injection. Cumulus cells surrounding MII oocytes were removed by 0.1% hyaluronidase treatment. Embryos were obtained from mated females during the following periods after hCG administration: 1-cell (18–20 hours), 2-cell (44–46 hours), 4-cell (54–56 hours), 8-cell (68–70 hours), morula (80–85 hours), and blastocyst (96–98 hours) [22].

### Microinjection, ICSI and in vitro Culture

Microinjections were performed in M2 medium. MII stage oocytes and 1-cell stage embryos were microinjected with *Pthlh* siRNA (sc-39696, Santa Cruz, Fig. 2B, Fig. S4). MII stage oocytes were performed intracytoplasmic sperm injection (ICSI) 3 h post-siRNA microinjection, a single sperm head was microinjected into the MII oocyte assisted with a Piezo-drill micromanipulator. Oocytes and embryos injected with control siRNA (GenePharma) were used as sham control. All injected embryos and uninjected control embryos were cultured in KSOM^AA^ (Millipore) following microinjection for 4 days in a 37°C, 5% CO_2_ atmosphere.

### RNA Isolation, Reverse-transcriptase and Quantitative Real-time Polymerase Chain Reaction (PCR)

Total RNA was isolated from 40 oocytes or preimplantation embryos of the injection groups and the control groups at the different developmental stages using the RNeasy Micro kit (74004, Qiagen) following the manufacturer’s manual. Oocytes and preimplantation embryos were transferred to lysis buffer and treated with the RNase-free DNase supplied in the kit. The mRNA was reverse-transcribed into cDNA using oligo (dT) primers and the PrimeScript™ 1^st^ strand cDNA synthesis kit (D6110, TaKaRa) according to the manufacturer’s instructions.

To measure mRNA levels, real-time RT-PCR analyses were performed using the ABI Prism 7500 real-time PCR system (Applied Biosystems). SYBR Premix Ex Taq™ reagents (DRR041, TaKaRa) were used for monitoring amplification, and the results were evaluated with the 7500 software program (v 2.0.1, Applied Biosystems). Real-time PCR primers were designed using the PrimerExpress software (Applied Biosystems) purchased from Invitrogen. All primers are shown in Table 4. The reaction mixture had a final volume of 20 µl and contained 10 µl SYBR Premix Ex Taq™ (2×), 0.4 µl forward primer (10 µM), 0.4 µl reverse primer (10 µM), 0.4 µl ROX Reference Dye (50×), 2.0 µl cDNA and 6.8 µl water. The PCR thermal cycling parameters were 95°C for 10 s and 40 cycles of 5 s at 95°C and 34 s at 60°C. Following the PCR reaction, the specificity of amplification for each gene was evaluated by monitoring the dissociation (melting) curve. Expression levels of different transcripts were normalized to the housekeeping gene *Gapdh* within the log-linear phase of the amplification curve using the ΔΔCt method.

**Table 4:** Primer sequences used for real-time PCR.

Gene name	Accession no.	Primer sequence (5′- 3′)	Annealing temperature (°C)	Product size (bp)
*Pthlh*	NM_008970	For-AATGCATTGGGATCAAACTGTCT	60	75
		Rev-GCCTTGGCAAAAGGGAAAA		
*Pth1r*	NM_011199	For-GCTTCCTGCCACCACCAAT	60	103
		Rev-GGGAACTGTCATAGTAACTGG		
*Nanog*	NM_028016	For- CCTGATTCTTCTACCAGTCCCA	60	123
		Rev-GGCCTGAGAGA ACACAGTCC		
*Pou5f1*	NM_013633	For- TGTTCCCGTCACTGCTCTGG	60	82
		Rev-TTGCCTTGGCTCACAGCATC		
*Gapdh*	NM_008084	For-TGGCAAAGTGGAGATTGTTGCC	60	156
		Rev-AAGATGGTGATGGGCTTCCCG		

### Western Blot Analysis

350 mouse embryos were collected in SDS sample buffer and heated for 5 min at 100°C. The proteins were subjected to 12% SDS-PAGE and transferred onto a polyvinylidene difluoride (PVDF) membrane (Amersham Biosciences, Piscataway, NJ). The membrane was blocked for 2 h in Tris-buffered saline-Tween (TBST) containing 5% nonfat dry milk at room temperature. The blocked membranes then were incubated overnight at 4°C with affinity-purified goat polyclonal anti-PTHLH antibody (1∶500; Santa Cruz) or mouse monoclonal anti-GAPDH antibody (1∶1,000, Zhong Shan Jin Qiao) in TBST. After washing three times in TBST with each time for 10 minutes, the membrane was incubated for 1 hour at 37°C with peroxidase-conjugated rabbit anti-goat IgG (1∶1,000, Zhong Shan Jin Qiao) or peroxidase-conjugated rabbit anti-mouse IgG (1∶1,000, Zhong Shan Jin Qiao), respectively. Finally, the membrane was processed using the SuperSignal West Femto maximum sensitivity substrate (Thermo Scientific).

### Synthesis of *Pthlh* mRNA and PTHLH (1–141) Protein

The PTHLH (1–141) gene fragment was amplified by PCR with the following primers: F: GCGAATTCTATGCTGCGGAGGCTGGTTCT, R: GCCTCGAGTCGCTTCTTTTTCTCCTGTT, and inserted into corresponding sites of expression vector pET-21b, and the fusion protein expressed in E.coli BL21 induced by IPTG. The purification of the fusion protein was used Ni-NTA His bind Resin (NOVAGEN) following the manufacturer’s manual. After purification, the target protein loaded dialysis bag using PBS to remove the ions, and then was concentrated by polyethylene glycol.

The embryos injected with *Pthlh* siRNA and the control embryos were cultured in KSOM^AA^ with or without 10 ng/ml PTHLH (1–141) [9] for 4 days following microinjection in a 37°C, 5% CO_2_ atmosphere.

### Immunofluorescence and Confocal Microscopy

All steps were performed at room temperature unless otherwise noted. Collected embryos were fixed with 4% paraformaldehyde for 30 min and permeabilized for 30 min with 0.2% Triton X-100 in PBS. Following three washes, all samples were incubated in a blocking solution (1% BSA and 0.05% Tween-20 in PBS) for at least 1 h. Embryos were then incubated with antibodies to H3K9 acetylation (1∶250, Upstate), H3K14 acetylation (1∶250, Upstate), NANOG (1∶500, Cosmo Bio) or OCT4 (1∶500, Santa Cruz) overnight at 4°C. Following three washes, embryos were incubated with a fluorescein isothiocyanate (FITC)-conjugated rabbit anti-mouse second antibody (1∶100; Zhong Shan Jin Qiao) or goat anti-rabbit second antibody (1∶100; Zhong Shan Jin Qiao) for 1 h. Following three washes in washing buffer, DNA was stained with Hoechst 33342 (10 µg/ml), and all samples were mounted in anti-fade solution. Stained and slide-mounted embryos were observed on a Zeiss LSM 710 microscope (Carl Zeiss, Germany). The same instrument settings were used for samples of the same development stage. Nuclear intensities of integrated fluorescence were measured with the ImageJ software (image processing and analysis in Java, http://rsb.info.nih.gov/ij/) as previously described [23], [24]. All individual nuclei in embryos at the 2-cell, 4-cell, 8-cell, 12 nuclei per morula and 20 nuclei per blastocyst stages were outlined manually. Following background subtraction, total fluorescence intensities of all individual nuclei in embryos at the 2-cell, 4-cell, 8-cell, 12 nuclei per morula and 20 nuclei per blastocyst stages were captured. For quantification, the ratios of the fluorescence intensity of OCT4, NANOG, acetylated histone H3-lysine 9 (Ac-H3K9) or acetylated histone H3-lysine 14 (Ac-H3K14) and that of Hoechst 33342 DNA signal were compared. All collected images were assembled using the Adobe Photoshop software (Adobe Systems, San Jose, CA) without contrast or brightness adjustments. At least five embryos at every developmental stage were processed for each experiment and the experiments were replicated at least three times.

### Cell Number Counting at the Blastocyst Stage

Cell numbers were determined as previously described for blastocysts cultured 90 h following microinjection [25]. Briefly, acidic Tyrode’s solution was used to remove the zona pellucida of blastocyst stage embryos. Zona-free embryos were then exposed to 10% (v/v) rabbit anti-mouse whole serum for 30 min at 37°C, and then incubated in a 10% (v/v) guinea pig complement with propidium iodide (PI) and Hoechst 33342 for 15 min at room temperature. Embryos were then immediately examined using an inverted NIKON fluorescence microscope (TE 200, Japan). Cells were counted directly under the microscope.

### Statistical Analysis

Statistical analyses of real-time PCR data, total cell number of blastocyst stageembryos and fluorescence intensity values were evaluated by one-way analysis of variance (ANOVA) with the SPSS 13.0 software. A value of P<0.05 was considered statistically significant.

## Supporting Information

Figure S1 **The effects of** ***Pthlh*** **siRNA injection on OCT4 and NANOG expression in mouse morula stage embryos.** Staining pattern of NANOG or OCT4 in the *Pthlh* siRNA-injected and the control embryos at the morula stage; OCT4/NANOG, green; DNA, blue. Bar = 20 µm.
(TIF)

Figure S2 **The effects of** ***Pthlh*** **siRNA injection on the acetylation of lysine 9 on histone H3 (Ac-H3K9) in mouse preimplantation embryos.** The staining pattern of Ac-H3K9 in the *Pthlh* siRNA-injected and the control siRNA-injected preimplantation embryos at the 2-cell stage; the 4-cell stage; the 8-cell stage; the morula stage and the blastocyst stage. Ac-H3K9, green; DNA, blue. Bar = 20 µm.
(TIF)

Figure S3 **The effects of** ***Pthlh*** **siRNA injection on the acetylation of lysine 12 on histone H3 (Ac-H3K14) in mouse preimplantation embryos.** The staining pattern of Ac-H3K14 in the *Pthlh* siRNA-injected and the control siRNA-injected preimplantation embryos at the 2-cell stage; the 4-cell stage; the 8-cell stage; the morula stage and the blastocyst stage. Ac-H3K14, green; DNA, blue. Bar = 20 µm.
(TIF)

Figure S4 **The effects of** ***Pthlh*** **siRNA injection on the expression of** ***Pthlh*** **in mouse 2-cell stage embryos.** Quantitative real-time PCR was applied to detect the expression of *Pthlh* in 2-cell embryos. Transcripts levels were normalized against *Gapdh* expression. 1: control siRNA-injected group; 2: *Pthlh* siRNA (sc-39696a)-injected group; 3: *Pthlh* siRNA (sc-39696b)-injected group; 4: *Pthlh* siRNA (sc-39696c)-injected group.
(TIF)

Table S1 Effect of *Pthlh* siRNA on development of embryos from mice in nature oestrus.
(DOC)
