# Supplementary figures and images for: The Roles of Parathyroid Hormone-Like Hormone during Mouse Preimplantation Embryonic Development
Source: PLoS One. 2012 Jul 13;7(7):e40528. doi: 10.1371/journal.pone.0040528 (PMC3396650; doi:10.1371/journal.pone.0040528)

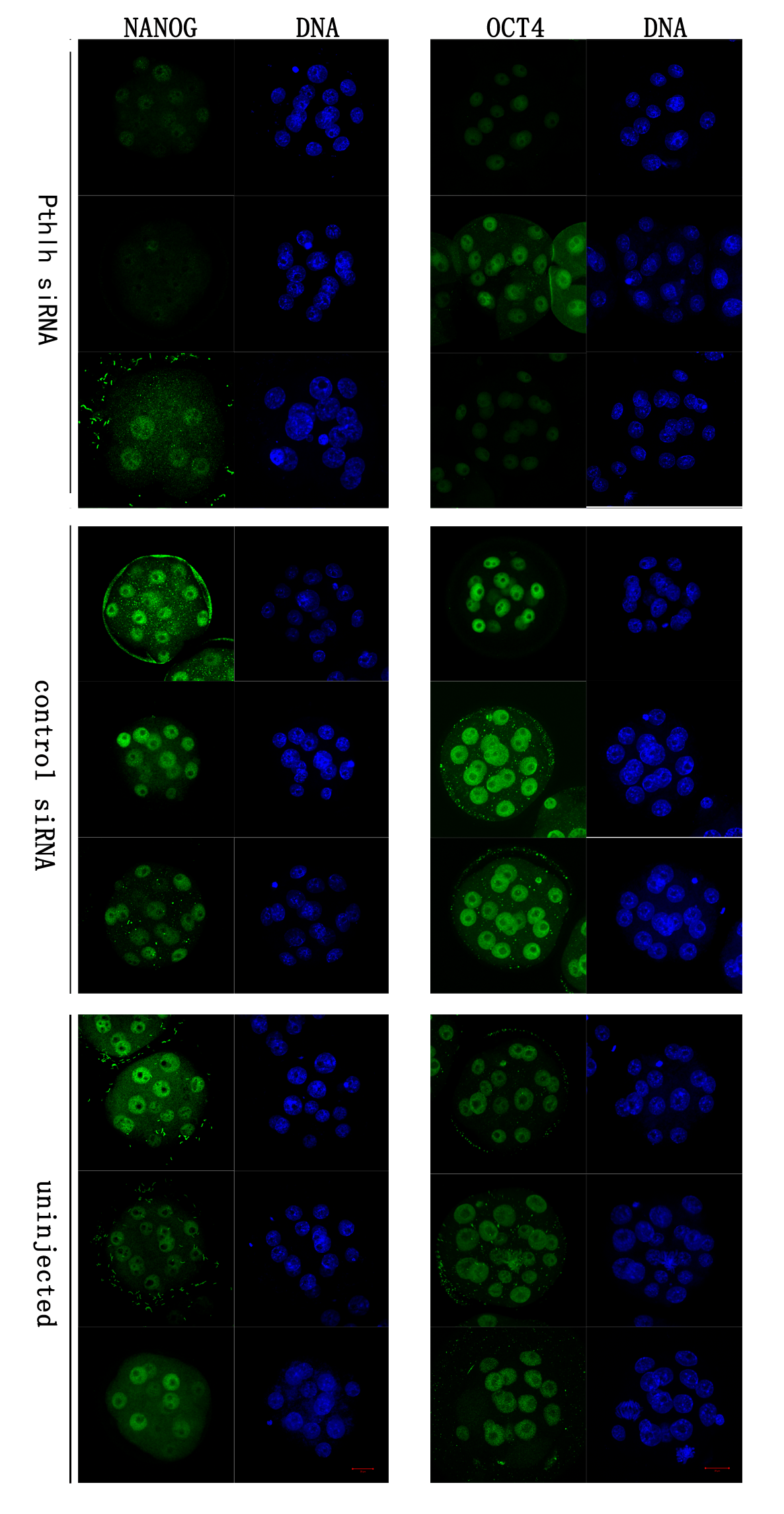

Supplement: Figure S1 — The effects of Pthlh siRNA injection on OCT4 and NANOG expression in mouse morula stage embryos. Staining pattern of NANOG or OCT4 in the Pthlh siRNA-injected and the control embryos at the morula stage; OCT4/NANOG, green; DNA, blue. Bar = 20 µm. (TIF) [file pone.0040528.s001.tif]

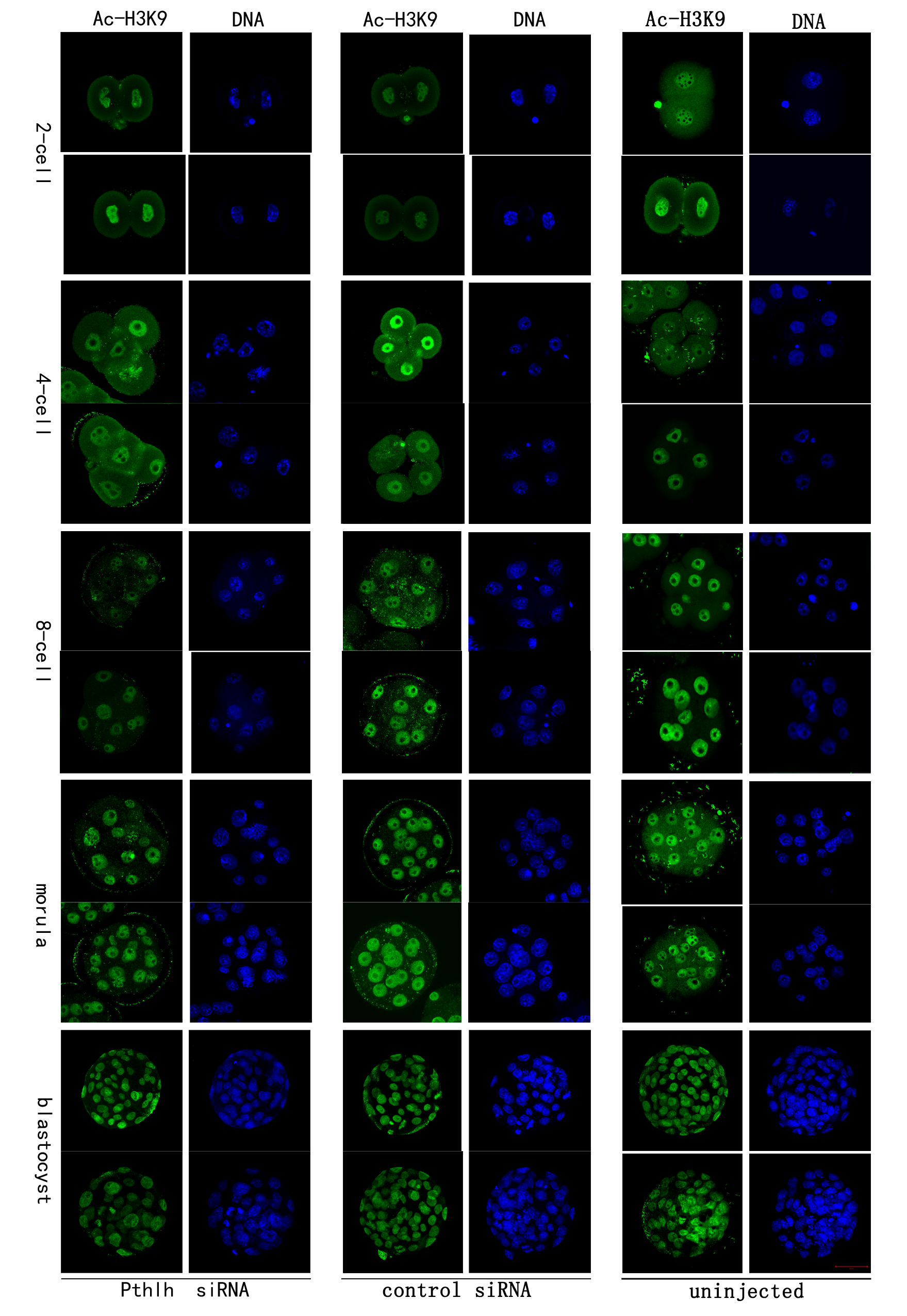

Supplement: Figure S2 — The effects of Pthlh siRNA injection on the acetylation of lysine 9 on histone H3 (Ac-H3K9) in mouse preimplantation embryos. The staining pattern of Ac-H3K9 in the Pthlh siRNA-injected and the control siRNA-injected preimplantation embryos at the 2-cell stage; the 4-cell stage; the 8-cell stage; the morula stage and the blastocyst stage. Ac-H3K9, green; DNA, blue. Bar = 20 µm. (TIF) [file pone.0040528.s002.tif]

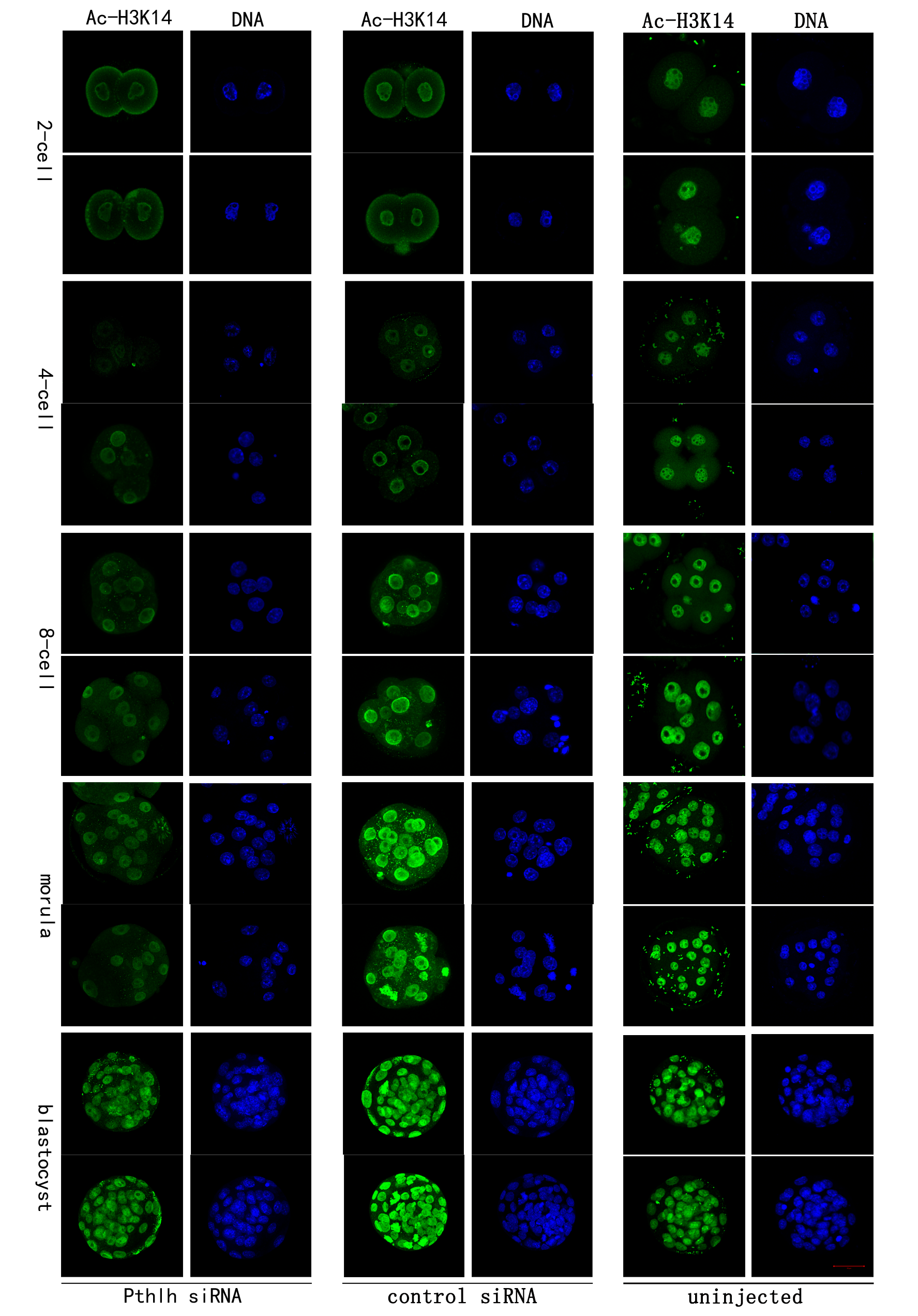

Supplement: Figure S3 — The effects of Pthlh siRNA injection on the acetylation of lysine 12 on histone H3 (Ac-H3K14) in mouse preimplantation embryos. The staining pattern of Ac-H3K14 in the Pthlh siRNA-injected and the control siRNA-injected preimplantation embryos at the 2-cell stage; the 4-cell stage; the 8-cell stage; the morula stage and the blastocyst stage. Ac-H3K14, green; DNA, blue. Bar = 20 µm. (TIF) [file pone.0040528.s003.tif]

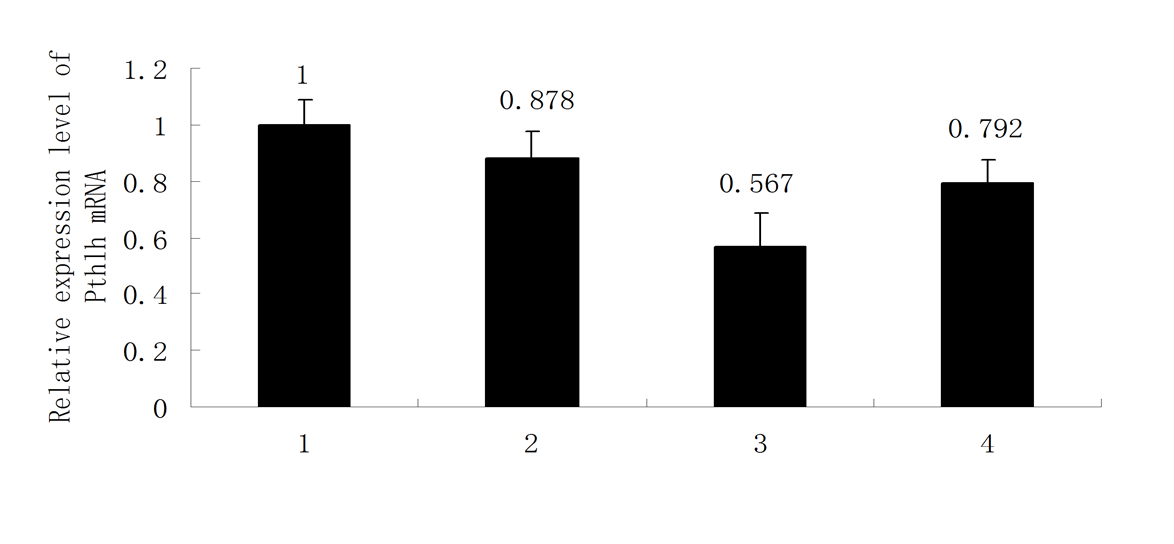

Supplement: Figure S4 — The effects of Pthlh siRNA injection on the expression of Pthlh in mouse 2-cell stage embryos. Quantitative real-time PCR was applied to detect the expression of Pthlh in 2-cell embryos. Transcripts levels were normalized against Gapdh expression. 1: control siRNA-injected group; 2: Pthlh siRNA (sc-39696a)-injected group; 3: Pthlh siRNA (sc-39696b)-injected group; 4: Pthlh siRNA (sc-39696c)-injected group. (TIF) [file pone.0040528.s004.tif]
