# Supplementary material for: The Roles of Parathyroid Hormone-Like Hormone during Mouse Preimplantation Embryonic Development
Source: PLoS One. 2012 Jul 13;7(7):e40528. doi: 10.1371/journal.pone.0040528 (PMC3396650; doi:10.1371/journal.pone.0040528)
Supplement: Table S1 — Effect of Pthlh siRNA on development of embryos from mice in nature oestrus. (DOC) [file pone.0040528.s005.doc]

**Table S1. Effect of *Pthlh* siRNA on development of embryos from mice in nature oestrus**

| Treatment | No. of embryos  (No. exp) | No. of blastocysts (%) |
| --- | --- | --- |
| uninjected | 40 (3) | 35 (87.5) |
| Control siRNA | 45 (3) | 39 (86.7) |
| *Pthlh* siRNA | 45 (3) | 16 (35.6)^*^ |

* p<0.01 compared with the control groups.
